# Supplementary material for: Identification of Late Pleistocene and Holocene fossil lizards from Hall’s Cave (Kerr County, Texas) and a primer on morphological variation in North American lizard skulls
Source: PLoS One. 2024 Aug 15;19(8):e0308714. doi: 10.1371/journal.pone.0308714 (PMC11326655; doi:10.1371/journal.pone.0308714)
Supplement: S2 Table — (DOCX) [file pone.0308714.s002.docx]

**S2 Table. Global-scale apomorphies taken from the existing literature and interpreted using a phylogenetic framework following Burbrink et al. [2020].**

*Denotes clades exhibiting known exceptions for an apomorphic feature

| Premaxilla |  |  |
| --- | --- | --- |
| **Apomorphy (context)** | **Character evolution**  **hypothesis** | **References** |
| Fused premaxilla (across Squamata) | Apomorphy of Squamata, independently lost in Scincidae* and Gekkota* | [1] |
| Less than seven tooth positions on the premaxilla (across Squamata) | Apomorphy of Iguania* | [2] |
| Incisive process absent (across Squamata) | Apomorphy of Lacertoidea* | [3–7] |
| Enlarged median tooth on the premaxilla (across Squamata) | Apomorphy of Dibamids and independently evolved in Amphisbaenia | [2,8,9] |
| Anterior premaxillary foramina (across Squamata) | Apomorphy of Iguania* and independently evolved in Anguimorpha*, Cordyloidea, and Amphisbaenia | [10,11] |
| Multicuspid premaxillary teeth (within Iguania) | Apomorphy of Iguanidae* | [11,12] |
| Nasal facets anteriorly on the nasal process (within Pleurodonta) | Apomorphy of Iguanidae*, independently evolved in Crotaphytidae and the clade (Liolaemidae, (Polychrotidae, (Hoplocercidae, (Opluridae, Leiosaurudae))))* | [13,14] |
| Anterior premaxillary foramina (within Phrynosomatidae) | Apomorphy of Phrynosomatinae within Phrynosomatidae and also occurs in *Uta** | [11] |
| Anterior rostral face flat and flush with nasal process (within Phrynosomtidae) | Apomorphy of Phrynosomatinae | [11] |
| Rectangular alveolar plate (within Phrynosomatidae) | Apomorphy of sand-lizard clade | [11] |
| Nasal process directed dorsally (within Phrynosomtidae) | Apomorphy of *Phrynosoma* | [11] |
| Base of nasal process nearly the same width and alveolar plate (within Phrynosomtidae) | Apomorphy of *Phrynosoma* | [11] |
| Forked palatal process (within Anguimorpha) | Apomorphy of Anguinae and Diploglossinae | [15–18] |
| Dorsal ossification on the alveolar plate posterior to medial ethmoidal foramen (within Anguidae) | Apomorphy of Diploglossinae and independently evolved in Gerrhonotinae | [18] |
| Ossified bridge extending laterally from nasal process that encloses the medial ethmoidal foramen (within Anguidae) | Apomorphy of Gerrhonotinae* | [19] |

| Maxilla |  |  |
| --- | --- | --- |
| **Apomorphy (context)** | **Character evolution hypothesis** | **References** |
| Elongate depression on the palatal shelf (gutter) encompassing the superior alveolar nerve and maxillary artery (across Squamata) | Apomorphy of Iguania* | [2,20] |
| Foramina for both the subnarial artery and the anterior inferior alveolar nerve on the premaxillary process (across Squamata) | Apomorphy of Pleurodonta* | [2] |
| Deep jugal groove on the postorbital process (within Pleurodonta) | Apomorphy of the clade (Crotaphytidae, (Corytophanidae, Leiocephalidae)) and independently evolved in Iguanidae and Opluridae | [2,21] |
| Flat dorsal surface of the palatine process (within Pleurodonta) | Apomorphy of Phrynosomatidae and also present in Leiolemidae, Tropiduridae, Anolidae, Polychrotidae, and Hoplocercidae | [2,21] |
| Large sub-triangular palatine process (within Pleurodonta) | Apomorphy of Phrynosomatidae and also present in Tropiduridae, Crotaphytidae, Leiocephalidae*, and Opluridae | [2,21] |
| Medially folded facial process with distinct canthal crest and anterodorsal facing surface (within Pleurondonta) | Apomorphy of Phrynosomatidae* and also present in Tropiduridae and *Anolis* | [2] |
| Narrow, triangular facial process (within Pleurodonta) | Apomorphy of *Phrynosoma** | [21,22] |
| Strongly asymmetric palatine process (within Pleurodonta) | Apomorphy of *Phrynosoma** | [2] |
| Tall, prominent crista transversalis (within Phrynosomatidae) | Apomorphy of *Phrynosoma** | [21] |
| Elongate premaxillary process with a low, anteromedially trending crista transversalis (within Anguidae) | Apomorphy of Gerrhonotinae | [10] |
| Deeply notched premaxillary process (within Anguidae) | Apomorphy of Diploglosinae and also present in Anguinae | [15] |

| Nasal |  |  |
| --- | --- | --- |
| **Apomorphy (context)** | **Character evolution hypothesis** | **References** |
| Distinct supranarial process (across Squamata) | Apomorphy of Iguania* | [9] |

| Frontal |  |  |
| --- | --- | --- |
| **Apomorphy (context)** | **Character evolution hypothesis** | **References** |
| Fused frontal (across Squamata) | Apomorphy of Gekkota independently evolved in Gymnophthalmoidea, Iguania, and some members of Anguimorpha* and Scincidae* | [23,24] |
| Strongly waisted interorbital margins of the frontal (across Squamata) | Apomorphy of Pleurodonta* independently evolved in some xenosaurs, gerrhonotines, and members of Gymnophthalmoidea* | [5,16,23] |
| Reduced descending cristae cranii of the frontal (across Squamata) | Apomorphy of Iguania independently evolved in Gymnophthalmoidea* | [16,23,25] |
| Parietal foramen partially or fully within the frontal (across Squamata) | Apomorphy of Iguania* | [12,26–28] |
| Co-ossified osteoderms on dorsal surface (across Squamata) | Apomorphy of Anguimorpha*  independently evolved in Scincomorpha* and Lacertidae* | [16,23] |
| Frontal tabs (across Squamata) | Apomorphy of Gymnphthalmoidea* independently evolved in Chamaeleonidae | [16,23] |

| Parietal |  |  |
| --- | --- | --- |
| **Apomorphy (context)** | **Character evolution hypothesis** | **References** |
| Fused parietal (across Squamata) | Apomorphy of Squamata* | [1,23] |
| Long ventrally projecting parietal crests or processes (across Squamata) | Apomorphy of Scincomorpha* independently evolved in Gymnophthalmoidea* and Anguimorpha | [16,23] |
| Long posterior projections (median extensions of Evans 2008) on the posterior edge of parietal table between the postparietal processes (across Squamata) | Apomorphy of Scincomorpha* | [9,16] |
| Co-ossified osteoderms on dorsal surface (across Squamata) | Apomorphy of Anguimorpha*  independently evolved in Scincidae* | [16,23] |
| Parietal foramen not fully enclosed by the parietal (across Squamata) | Apomorphy of (Gekkota, Dibamidae) independently evolved in Amphisbaenia*, Gymnophthalmoidea*, Helodermatidae, and Pleurodonta* | [23] |
| Parietal lappets (across Squamata) | Apomorphy of Lacertoidea* independently evolved in Xantusiidae and some iguanians, cordylids, and scincids | [23] |

| Prefrontal |  |  |
| --- | --- | --- |
| **Apomorphy (context)** | **Character evolution hypothesis** | **References** |
| Prefrontal boss (across Squamata) | Apomorphy of Iguania* also present in Teiinae | [9,10,23] |
| Well developed lateral projection buttressing the lacrimal (across Squamata) | Independently evolved apomorphy of Gymnophthalmoidea, Corytophanidae, and Scincidae | [2,29] |
| Lacrimal fused to prefrontal (across Squamata) | Independently evolved apomorphy of Xantusiidae, Gymnopthamoidea*, and Amphisbaenia | [16,23,25,30] |

| Jugal |  |  |
| --- | --- | --- |
| **Apomorphy (context)** | **Character evolution hypothesis** | **References** |
| Quadratojugal process absent (across Squamata) | Apomorphy of Iguania* | [1,2] |
| Angulated jugal (across Squamata) | Apomorphy of Unidentata* (sensu Burbrink et al. 2020), lost in Iguania* | [4] |
| Medial ectopterygoid process (across Squamata) | Apomorphy of Lacertoidea* | [16] |
| Medial ridge located at the midline of the suborbital and postorbital processes (across Squamata) | Apomorphy of Anguimorpha* | [31] |
| Wide postorbital process in lateral view (across Squamata) | Apomorphy of Xenosauridae independently evolved in some iguanians, and Xantusiidae | [2,9,30] |
| Posteriorly deflected distal end of the postorbital process (within Pleurodonta) | Apomorphy of Phrynosomatidae independently evolved in Tropiduridae, Crotaphytidae, and Opluridae | [2] |
| Long quadratojugal process (within Anguidae) | Apomorphy of Gerrhonotinae* independently evolved in some diploglossines | [32,33] |

| Postfrontal |  |  |
| --- | --- | --- |
| **Apomorphy (context)** | **Character evolution hypothesis** | **References** |
| Not elongated in mediolateral or anteroposterior plane (across Squamata) | Apomorphy of Iguania* | [2,4] |

| Postorbital |  |  |
| --- | --- | --- |
| **Apomorphy (context)** | **Character evolution hypothesis** | **References** |
| Postorbital forms more than half of orbital border and has a distinct ventral process (across Squamata) | Apomorphy of Iguania* | [23] |
| Anteriorly projecting supraorbital spine (across Squamata) | Apomorphy of Corytophanidae independently evolved in *Phrynosoma* | [2] |
| Fused postorbitofrontal (across Squamata) | Apomorphy of Xantusiidae independently evolved in Xenosauridae*, Lacertoidea*, some anguimorphs*, and some scincids* | [4,23] |
| Dorsal facet for frontoparietal articulation on dorsal process (within Iguania) | Apomorphy of Crotaphytidae also occurs in Corytophanidae, Polychrotidae, Anolidae*, and Leiosauridae | Adapted from [2] |
| Convex dorsal margin of posterior process (within Iguania) | Apomorphy of Corytophanidae independently evolved in some oplurids, *Leiocephalus*, and agamids | [2] |
| Lateral tubercle mid-height on the postorbital (within Pleurodonta) | Apomorphy of Crotaphytidae independently evolved in Tropiduridae and Opliuridae* | [2] |

| Squamosal |  |  |
| --- | --- | --- |
| **Apomorphy (context)** | **Character evolution hypothesis** | **References** |
| Posterodorsal process (across Squamata) | Apomorphy of Iguania* independently evolved in some clades within Teiidae and Xenosauridae | [2,16,23,34] |

| Quadrate |  |  |
| --- | --- | --- |
| **Apomorphy (context)** | **Character evolution hypothesis** | **References** |
| Notch or foramen on dorsal surface for articulation with the squamosal (across Squamata) | Apomorphy of Squamata | [1] |
| Distinct pterygoid lappet present (across Squamata) | Apomorphy of Lacertoidea* and independently evolved in Helodermatidae, Xenosauridae, Scincidae*, and Iguania* | [16,23] |
| Low ridge on the posterior surface of the conch (within Iguania) | Apomorphy of Corytophanidae independently evolved in some members of Hoplocercidae, Anolidae, Polychrotidae, Phrynosomatidae, and Agamidae | [2] |

| Pterygoid |  |  |
| --- | --- | --- |
| **Apomorphy (context)** | **Character evolution hypothesis** | **References** |
| Absence of pterygoid tooth loci (across Squamata) | Apomorphy of (Gekkota, Dibamidae), independently evolved in Xantusiidae, Amphisbaenia, and some clades within Anguimorpha* and Iguania* | [23] |
| Well-developed ventromedial projection at the floor of the basipterygoid fossa (across Squamata) | Apomorphy of Iguania* independently evolved in some clades within Anguimorpha*  –alternatively an apomorphy of Toxicodera lost in some clades | [10,16] |

| Ectopterygoid |  |  |
| --- | --- | --- |
| **Apomorphy (context)** | **Character evolution hypothesis** | **References** |
| Elongate posterolateral process (across Squamata) | Apomorphy of Iguania independently evolved in Xantusiidae and Xenosauridae  –alternatively a plesiomorphy of Squamata retained in these clades | [10] |
| Broad ectopterygoid (across Squamata) | Apomorhy of Teiidae* independently evolved in Xantusiidae*, Amphisbaenia, and Dibamidae | [9] |

| Marginal Dentition |  |  |
| --- | --- | --- |
| **Apomorphy (context)** | **Character evolution hypothesis** | **References** |
| Pleurodont teeth (i.e. tooth position lingual relative to labial wall of tooth bearing bone) (across Squamata) | Apomorphy of Lepidosauramorpha* | [35] |
| Tricuspid teeth (across Squamata) | Apomorphy of Pleurodonta independently evolved within Xantusiidae and Gymnophthalmoidea* | [21,29,30,34] |
| Asymmetrically bicuspid distal teeth (across Squamata) | Apomorphy of Lacertoidea* | [36] |
| Large amount of cementum deposits at base of teeth (across Squamata) | Apomorphy of Teiidae | [23,36,37] |
| Unicuspid teeth with striated crowns (across Squamata) | Apomorphy of Scincomorpha independently evolved within Anguimorpha | [10,38] |
| Tooth bases much wider than the crowns (within Pleurodonta) | Apomorphy of Anolidae independently evolved in Crotaphytidae | [10,13] |

| Dentary |  |  |
| --- | --- | --- |
| **Apomorphy (context)** | **Character evolution hypothesis** | **References** |
| Meckelian groove open ventrally, anterior to the inferior alveolar foramen (across Squamata) | Apomorphy of Scincidae* and independently evolved within Gymnophthalmoidea*, Anguimorpha, and some chamaeleonids  –alternatively an apomorphy of Unidentata lost in Iguania* and Cordyloidea* | [5,16,23,36,39,40] |
| Inframeckelian lip curls dorsolingually, producing a medial exposure of the Meckelian groove along the mid-length of the dentary (across Squamata) | Apomorphy of Iguania* and independently evolved within some teiids and Cordyloidea* | [9,16,40,41] |
| Suprameckelian and inframeckelian lips constrict or close Meckelian groove (across Squamata) | Apomorphy of Iguania* and independently evolved within some scincids and teiids | [9,21,41,42] |
| Suprameckelian and inframeckelian lips indistinguishably fused, enclosing the Meckelian groove (across Squamata) | Apomorphy of (Gekkota, Dibamidae) independently evolved in Amphisbaenians, Xantusiidae, Gymnophthalmoidea*, Pleurodonta, and some scincids | [9,16,21,23,28,42,43] |
| Broad subdental shelf (across Squamata) | Apomorphy of (Gekkota, Dibamidae), and independently evolved in Scincomorpha, and Lacertoidea*  –alternatively small subdental shelf is an apomorphy of Toxicofera | [23] |
| Surangular notch or presence of surangular process (across Squamata) | Apomorphy of Anguimorpha* | [23,44,45] |
| Intramandibular septum divides Meckel’s canal near the posterior end of tooth row (across Squamata) | Apomorphy of Anguimorpha independently evolved in some clades within Iguania* | [23,46] |
| Splenial spine (across Squamata) | Apomorphy of Anguimorpha* | [4,47] |
| Well developed intramandibular lamella (across Squamata) | Apomorphy of Teiidae* independently evolved in some clades within Pleurodonta* | [2,36,48] |
| Elongate Meckelian groove providing space for hypertrophied splenial (across Squamata) | Apomorphy of Teiidae | [36] |
| Large incision between the coronoid and angular processes (within Teiidae) | Apomorphy of Tupinambinae | [36] |
| Suprameckelian and inframeckelian lips indistinguishably fused, enclosing the Meckelian groove (within Pleurodonta ) | Apomorphy of Iguanidae, Anolidae, Tropiduridae, Leiocephalidae, Polychrotidae, Leiosaurudae*, and some members of Opluridae, Corytophanidae, and Phrynosomatidae | [21,28,43] |
| Intramandibular septum with free posteroventral margin (within Anguimorpha) | Apomorphy of Anguidae* | [17,44] |
| Intramandibular septum fused to posterior inner wall of dentary (within Anguidae) | Apomorphy of Anguinae, also occurs in Diploglossinae | [46,49,50] |
| Surangular spine (within Anguidae) | Apomorphy of *Ophisaurus* | [49] |

| Coronoid |  |  |
| --- | --- | --- |
| **Apomorphy (context)** | **Character evolution** | **References** |
| Distinct coronoid process formed only by coronoid (across Squamata) | Apomorphy of Squamata | [1] |
| Distinct anteriorly projecting lateral process (across Squamata) | Apomorphy of Squamata, lost in Xantusiidae and some clades within Iguania* | [23] |

| Splenial |  |  |
| --- | --- | --- |
| **Apomorphy (context)** | **Character evolution** | **References** |
| Anterior inferior foramen enclosed by splenial (across Squamata) | Apomorphy of Gymnophthalmoidea* independently evolved in some clades within Iguania*, in Scincomorpha*, and some clades within Anguimorpha* | [5,9,16] |
| Anterior inferior alveolar foramen posterodorsal to anterior mylohyoid foramen (across Squamata) | Apomorphy of Gymnophthalmoidea* | [9] |
| Splenial fused to dentary (across Squamata) | Apomorphy of Xantusiidae | [9] |

| Compound bone |  |  |
| --- | --- | --- |
| **Apomorphy (context)** | **Character evolution hypothesis** | **References** |
| Condyle formed only by articular (across Squamata) | Apomorphy of Lepidosauria | [1] |
| Medial inflection of retroarticular process (across Squamata) | Apomorphy of Scincomorpha independently evolved in Anguimorpha and Gekkota | [23] |
| Lack of oblique torsion of retroarticular process (across Squamata) | Apomorphy of Lactertoidea independently evolved in Pygopodidae and Iguania* | [23] |
| Tubercle or flange on posteromedial margin of retroarticular process (across Squamata) | Apomorphy of Scincidae | [23] |
| Notch on the medial margin of the retroarticular process (across Squamata) | Apomorphy of Gekkota | [23] |
| Distinct angular process (across Squamata) | Apomorphy of Iguania* and independently evolved in Gymnophthalmoidea | [2,23] |
| Anterior surangular foramen ventrally located on surangular (across Squamata) | Apomorphy of Acrodonta independently evolved in Hoplocercidae and some members of Iguanidae | [2,12,51] |
| Broadened retroarticular process (across Squamata) | Apomorphy of Anguimorpha independently evolved in Gekkota and Scincomorpha* | [23] |
| Widely open adductor fossa (across Squamata) | Apomorphy of Gymnophthalmoidea independently evolved in some clades within Scincidae | [23] |
| Prearticular crest present (across Squamata) | Apomorphy of Gymnophthalmoidea independently evolved in Xantusiidae | [9] |

**References**

1. Gauthier J, Estes R, De Queiroz K. A phylogenetic analysis of Lepidosauromorpha. Phylogenetic relationships of the lizard families. Stanford University Press Stanford; 1988. pp. 15–98.

2. Smith KT. Eocene Lizards of the Clade *Geiseltaliellus* from Messel and Geiseltal, Germany, and the Early Radiation of Iguanidae (Reptilia: Squamata). Bulletin of the Peabody Museum of Natural History. 2009;50: 219–306. doi:10.3374/014.050.0201

3. Gao K, Norell MA. Taxonomic Revision of *Carusia* (Reptilia: Squamata) from the Late Cretaceous of the Gobi Desert and Phylogenetic Relationships of Anguimorphan Lizards. American Museum Novitates. 1998;3230: 1–52.

4. Conrad JL. Phylogeny and systematics of Squamata (Reptilia) based on morphology. Bulletin of the American Museum of Natural History. 2008;310: 1–182.

5. Hernández Morales C, Peloso PLV, Bolívar García W, Daza JD. Skull Morphology of the Lizard *Ptychoglossus vallensis* (Squamata: Alopoglossidae) With Comments on the Variation Within Gymnophthalmoidea. Anat Rec. 2019;302: 1074–1092. doi:10.1002/ar.24038

6. Villa A, Delfino M. A comparative atlas of the skull osteology of European lizards (Reptilia: Squamata). Zoological Journal of the Linnean Society. 2019;187: 829–928. doi:10.1093/zoolinnean/zlz035

7. Gans C, Montero R. An atlas of amphisbaenian skull anatomy. Biology of the Reptilia. 2008. pp. 621–738.

8. Lee MSY. Convergent evolution and character correlation in burrowing reptiles: towards a resolution of squamate relationships. Biological Journal of the Linnean Society. 1998;65: 369–453. doi:10.1111/j.1095-8312.1998.tb01148.x

9. Gauthier JA, Kearney M, Maisano JA, Rieppel O, Behlke ADB. Assembling the Squamate Tree of Life: Perspectives from the Phenotype and the Fossil Record. Bulletin of the Peabody Museum of Natural History. 2012;53: 3–308. doi:10.3374/014.053.0101

10. Smith KT. A new lizard assemblage from the earliest Eocene (Zone Wa0) of the Bighorn Basin, Wyoming, USA: Biogeography during the warmest interval of the Cenozoic. Journal of Systematic Palaeontology. 2009;7: 299–358. doi:10.1017/S1477201909002752

11. Scarpetta SG. The first known fossil *Uma*: ecological evolution and the origins of North American fringe-toed lizards. BMC Evol Biol. 2019;19: 178. doi:10.1186/s12862-019-1501-5

12. de Queiroz K. Phylogenetic Systematics of iguanine lizards. University of California Publications in Zoology. 1987;118: xii + 1-203.

13. Etheridge R, de Queiroz K. A phylogeny of Iguanidae. In: Estes R, Pregill GK, editors. Phylogenetic relationships of the lizard families. Stanford University Press Stanford, California; 1988. pp. 283–367.

14. Daza JD, Abdala V, Arias JS, García-López D, Ortiz P. Cladistic Analysis of Iguania and a Fossil Lizard from the Late Pliocene of Northwestern Argentina. Journal of Herpetology. 2012;46: 104–119. doi:10.1670/10-112

15. Meszoely CAM. North American fossil anguid lizards. Bulletin of the Museum of Comparative Zoology at Harvard College. 1970;139: 87–149.

16. Evans SE. The skull of lizards and tuatara. New York: Society for the Study of Amphibians and Reptiles; 2008.

17. Conrad JL, Ast JC, Montanari S, Norell MA. A combined evidence phylogenetic analysis of Anguimorpha (Reptilia: Squamata). Cladistics. 2011;27: 230–277. doi:10.1111/j.1096-0031.2010.00330.x

18. Scarpetta S. The earliest known occurrence of *Elgaria* (Squamata: Anguidae) and a minimum age for crown Gerrhonotinae: Fossils from the Split Rock Formation, Wyoming, USA. Palaeontologicia Electronica. 2018;21.1.1FC: 1–9. doi:0.26879/837

19. Scarpetta SG, Ledesma DT, Bell CJ. A new extinct species of alligator lizard (Squamata: *Elgaria*) and an expanded perspective on the osteology and phylogeny of Gerrhonotinae. BMC Ecology and Evolution. 2021;21: 184. doi:10.1186/s12862-021-01912-8

20. Smith KT. A diverse new assemblage of Late Eocene squamates (Reptilia) from the Chadron Formation of North Dakota. Palaeontologicia Electronica. 2006;9: 1–44.

21. Scarpetta SG. Iguanian lizards from the Split Rock Formation, Wyoming: exploring the modernization of the North American lizard fauna. Journal of Systematic Palaeontology. 2021;19: 221–251. doi:10.1080/14772019.2021.1894612

22. Bell CJ, Head JJ, Mead JI. Synopsis of the herpetofauna from Porcupine Cave. In: Barnosky AD, editor. Biodiversity Response to Climate Change in the Middle Pleistocene: the Porcupine Cave Fauna from Colorado. Berkeley, California: University of California Press; 2004. pp. 117–126.

23. Estes R, de Queiroz K, Gauthier J. Phylogenetic relationships within Squamata. In: Estes R, Pregill GK, editors. Phylogenetic relationships of the lizard families. Stanford University Press Stanford; 1988. pp. 119–281.

24. Greer AE. A subfamilial classification of scincid lizards. Bulletin of the Museum of Comparative Zoology at Harvard College. 1970;139: 151–183.

25. Presch W. Evolutionary History of the South American Microteiid Lizards (Teiidae: Gymnophthalminae). Copeia. 1980;1980: 36. doi:10.2307/1444132

26. Etheridge R. The Relationships Of The Anoles (Reptilia:Sauria:Iguanidae) An Interpretation Based On Skeletal Morphology. Ph.D. Dissertation, University of Michigan. 1959.

27. Etheridge R. The Skeletal Morphology and Systematic Relationships of Sceloporine Lizards. Copeia. 1964;1964: 610–631.

28. Smith KT. The Evolution of Mid-Latitude Faunas During the Eocene: Late Eocene Lizards of the Medicine Pole Hills Reconsidered. Bulletin of the Peabody Museum of Natural History. 2011;52: 3–105. doi:10.3374/014.052.0101

29. Bell C, Evans S, Maisano J. The skull of the gymnophthalmid lizard *Neusticurus ecpleopus* (Reptilia: Squamata). Zoological Journal of the Linnean Society. 2003;139: 283–304.

30. Savage JM. Studies on the lizard family Xantusiidae. IV. The genera. Contributions in science. 1963;71: 1–38. doi:10.5962/p.241019

31. Čerňanský A, Smith KT, Klembara J. Variation in the Position of the Jugal Medial Ridge Among Lizards (Reptilia: Squamata): Its Functional and Taxonomic Significance. The Anatomical Record. 2014;297: 2262–2272. doi:10.1002/ar.22989

32. Rieppel Olivier. The phylogeny of anguinomorph lizards. Basel, Boston: Birkhäuser Verlag; 1980.

33. Ledesma DT, Scarpetta SG, Bell CJ. Variation in the skulls of *Elgaria* and *Gerrhonotus* (Anguidae, Gerrhonotinae) and implications for phylogenetics and fossil identification. PeerJ. 2021;9: e11602. doi:10.7717/peerj.11602

34. Tedesco ME, Krause L, Alvarez BB. Descripción del sincraneo de *Ameiva Ameiva* ) (Linnaeus) (Squamata, Teiidae). Rev Bras Zool. 1999;16: 1025–1044. doi:10.1590/S0101-81751999000400013

35. Simões TR, Caldwell MW, Tałanda M, Bernardi M, Palci A, Vernygora O, et al. The origin of squamates revealed by a Middle Triassic lizard from the Italian Alps. Nature. 2018;557: 706–709. doi:10.1038/s41586-018-0093-3

36. Scarpetta SG. Unusual lizard fossil from the Miocene of Nebraska and a minimum age for cnemidophorine teiids. R Soc open sci. 2020;7: 200317. doi:10.1098/rsos.200317

37. Nydam RL, Eaton JG, Sankey J. New Taxa Of Transversely-Toothed Lizards (Squamata: Scincomorpha) And New Information On The Evolutionary History Of “Teiids.” Journal of Paleontology. 2007;81: 538–549. doi:10.1666/03097.1

38. Estes R. A new gerrhonotine lizard from the Pliocene of California. Copeia. 1963;1963: 676–680.

39. Nash DF. A comparative study of the head and thoracic osteology and myology of the skinks, *Eumeces gilberti* Van Denburgh and *Eumeces skiltonianus* (Baird and Girard). M.S. Thesis, Brigham Young University. 1970.

40. Lang M. Generic relationships within Cordyliformes (Reptilia . Squamata). Bulletin De L’institut Royal Des Sciences Naturelles De Belgique, Biologie. 1991;61: 121–188.

41. Bochaton C, Boistel R, Grouard S, Ineich I, Tresset A, Bailon S. Evolution, diversity and interactions with past human populations of recently extinct *Pholidoscelis* lizards (Squamata: Teiidae) from the Guadeloupe Islands (French West-Indies). Historical Biology. 2019;31: 140–156. doi:10.1080/08912963.2017.1343824

42. Greer A. The genetic relationships of the Scincid lizard genus *Leiolopisma* and its relatives. Aust J Zoo Supps. 1974;22: 1–67.

43. Mead JI, Roth EL, Van Devender TR, Steadman DW. The late Wisconsinan vertebrate fauna from Deadman Cave, southern Arizona. Transactions of the San Diego Society of Natural History. 1984;20: 247–276.

44. Gauthier JA. Fossil xenosaurid and anguid lizards from the early Eocene Wasatch Formation, Southeast Wyoming, and a revision of the Anguioidea. Rocky Mountain Geology. 1982;21: 7–54.

45. Good DA. The phylogenetic position of fossils assigned to the Gerrhonotinae (Squamata: Anguidae). Journal of Vertebrate Paleontology. 1988;8: 188–195. doi:10.1080/02724634.1988.10011697

46. Pregill GK. Late Pleistocene herpetofaunas from Puerto Rico. Miscellaneous publication - University of Kansas, Museum of Natural History. 1981;71: 1–72.

47. Čerňanský A, Augé ML. The Oligocene and Miocene fossil lizards (Reptilia, Squamata) of Central Mongolia. Geodiversitas. 2019;41: 811–839. doi:10.5252/geodiversitas2019v41a24

48. Denton RK, O’Neill RC. *Prototeius stageri* , Gen. et sp. Nov., a New Teiid Lizard from the Upper Cretaceous Marshalltown Formation of New Jersey, with a Preliminary Phylogenetic Revision of the Teiidae. Journal of Vertebrate Paleontology. 1995;15: 235–253. doi:10.1080/02724634.1995.10011227

49. Klembara J, Hain M, Dobiašová K. Comparative Anatomy of the Lower Jaw and Dentition of *Pseudopus apodus* and the Interrelationships of Species of Subfamily Anguinae (Anguimorpha, Anguidae): Anatomy of Lower Jaw and Teeth of Anguinae. Anat Rec. 2014;297: 516–544. doi:10.1002/ar.22854

50. Syromyatnikova E, Aranda E. A record of galliwasp (Diploglossidae: *Diploglossus*) from the Pleistocene of Cuba. Historical Biology. 2022; 1–5. doi:10.1080/08912963.2022.2077108

51. Frost DR, Etheridge R. A Phylogenetic analysis and taxonomy of iguanian lizards (Reptilia, Squamata). Laurence, Kansas: Miscellaneous publication (University of Kansas. Museum of Natural History); 1989. Available: https://www.biodiversitylibrary.org/item/55046
