# Supplementary material for: Identification of Late Pleistocene and Holocene fossil lizards from Hall’s Cave (Kerr County, Texas) and a primer on morphological variation in North American lizard skulls
Source: PLoS One. 2024 Aug 15;19(8):e0308714. doi: 10.1371/journal.pone.0308714 (PMC11326655; doi:10.1371/journal.pone.0308714)
Supplement: Attachment — Submitted filename: Response to Reviewers.docx [file pone.0308714.s007.docx]

Reviewer #1

Comment 1) The first of these suggestions is as much philosophical as it is at the root of the methodology and purpose of this manuscript. Throughout the manuscript the authors comment on the benefits of understanding "lizard" diversity and how changes in this diversity could greatly affect our views on changing population dynamics, specifically in localized extirpations and emigrations. However, snakes are not mentioned at all despite being deeply nested in Squamata and therefore is a "lizard". Two dozen or so snake species have currently been identified living in or around the region today so the omission of snake diversity within the scope of lizards is puzzling and leaves a large gap in understanding of lizard diversity through recent time (Pleistocene to present). If the fossils are simply not present due to a preservational bias, this needs to be mentioned. If there are snake fossils from this locality, they should be included - or clear justification for their omission should be provided.
           Response 1) While we acknowledge that it would be interesting and beneficial to examine snakes from Hall’s Cave to understand squamate diversity, we disagree that not including snakes precludes us from examining lizard diversity from this site. The term “lizard” describes a paraphyletic group and does not include snakes, whereas the clade Squamata includes lizards and snakes (de Queiroz and Gauthier 2020). Snakes have been described from Hall’s Cave (Parmley 1988) and we have added commentary in the manuscripts making this clear. Like many (if not most) squamate paleontologists, we found it useful in this paper to examine lizards separately, even though “lizards” are a grade–and snakes represent a highly diverse, and both ecologically and phenotypically divergent, clade within Squamata. More importantly, an examination of snakes from this site would require an equal, if not lengthier, description and photographic documentation of fossil and modern snakes. Given the already great length of this manuscript, we consider this to be far outside of the scope of the current study.
Comment 2) The second suggestion is regarding the phylogenetic framework for apomorphy assessments. In the table S2, evolutionary context is given for apomorphy appearance, reversals, independent acquisitions, etc. If these evolutionary claims are based off the phylogeny you have provided, that should be explicitly stated. This, however, comes with the caveat that these statements could be very different when using a different tree topology (e.g., Simoes et al., 2018). The phylogeny provided also does not include snakes, which are deeply nested in the Squamate clade. I recommend removing evolutionary statements such as number of aquisitions and/or losses, or reversals. These do not matter for diagnosing elements to a group using apomorphy based assessments and only serves to confuse the associations.
           Response 1) Our phylogenetic framework follows that of Burbrink et al. (2020) for apomorphies taken from the literature that we mention in the main text and in table S2. We have added additional text in the methods section and the caption of table S2 reinforcing this. This phylogeny does include all extant squamates and evolutionary hypotheses we make about global-scale apomorphies are based on this inclusive topology. For table S2, we specify that these are global-scale apomorphies taken from the existing literature meaning that these apomorphies do take into account hypothesized phylogenetic relationships among all squamates. In some cases where we are assessing apomorphies within a particular squamate clade (noted in parentheses in table S2 for each apomorphy) it is not necessary to examine the evolutionary history of those characters across all squamates. Hypotheses on character evolution are necessary for developing a framework for apomorphy-based identifications, and if there are several potential apomorphy scenarios then these should be stated explicitly. Although details on acquisitions and/or losses, or reversals are not always referenced when diagnosing fossils, that information is inseparable from the practice of making identifications using apomorphies. Hypotheses regarding character evolution are therefore always present, whether explicitly stated or not for apomorphy identifications. We feel it is important to include hypotheses about character evolution in order to be explicit about our thinking regarding character evolution.
Comment 3) The figures are beautiful and specimens are clearly imaged. However, labels are only provided on one figure and do not always reflect the apomorphic states you are trying to discuss. I think it would be beneficial to include labels on other figures, especially those of significant importance for diagnosing an element to one group or another. With the amount of jumping around between figures, this would save from having to also go back to the only two labeled figures and S2 all the time. To the same point, showing multiple aspects of the elements would be a huge benefit as well. There were many times I would have made a more precise, or different general referral based on the images you have provided (e.g., Ophisaurus and fossil anguine elements). Based on the single aspect and general morphology I found a number of elements near spot on for Ophisaurus, but the same was not said in the description. I think showing multiple aspects as well as labelling the apomorphic states would help alleviate false identifications made by a reader's limited view.
           Response 1) It was our intention to provide a couple of reference figures with labeled anatomical features so that the panels of skeletal elements did not become unintelligible with labels and lines. That said, we do acknowledge that adding some labels to the figures will save readers time without having to always refer to the reference figures. We added labels for relevant anatomical features on a few specimens in each figure panel.
           Response 2) To the second point on increasing the number of illustrated orientations of elements, we agree that this would be informative, but it would require doubling or tripling the number of (already numerous) figures and ultimately is not necessary for this study. To your specific point that some fossils resemble *Ophisaurus*, fossils were indeed identified to *Ophisaurus*, but we stated that we only made these identification on the basis of modern biogeography (*Ophisaurus* is the only extant anguine in North America). We could identify no apomorphies that could be used to differentiate those elements from all other anguines, and images from multiple orientations of the elements would not further illuminate this point. Furthermore, the reader will always have a limited view of a 3D object presented on a 2D medium, but the reader is guided by both the figures as well as the in text descriptions and corresponding identifications. If you have other identification discrepancies in mind please let us know.
Comment 4) The descriptions are done very well! Some concluding sentences in description paragraphs could be more specific to avoid ambiguity. Additionally, referral based on size of a feature (e.g., parietal horns in phrynosomatids) seem poor features to use for genus or species referrals without a relative statement or quantification of these differences. As presented I am unconvinced of some of the more specific referrals within the phrynosomatids and additional data with provided measurements or relative statemens that can be clearly demonstrated would greatly improve the support for these referrals.
           Response 1) We have changed the wording of concluding sentences to make them more specific and reemphasize the features that permit identification. We have also changed our wording on referrals based on size. Our referrals were not based on absolute size, but rather on relative size. The parietal horns of species of *Phrynosoma*, for example, are differentiated by their size relative to the size of the parietal table. Our figured specimens of *Phrynosoma cornutum*, *Phrynosoma modestum*, and *Phrynosoma douglasii* show the range of the differences in relative proportions of the horn length to the size of the parietal table. If you have specific identification comments on phrynosomatids besides the parietal horns of *Phrynosoma* (a highly diagnostic and apomorphic feature of that genus) then please let us know.

Lines 98-102: We updated the figure 1 caption stating that it shows the elements recovered as fossils and examined in the text highlighted in purple.

Lines 106-109: We have included text mentioning that snake fossils have been recovered and described from Hall’s Cave. We also added text mentioning that treatment of snake fossils using an apomorphy-based fossil identification framework awaits an equally lengthy discussion in future work.

Lines 125-129: In many cases, our global-scale apomorphy based identifications allowed us to identify fossils to clades with NA and non-NA components. In these cases, we geographically restricted our identifications on the continental scale. For example, in some cases, we could identify fossils using global-scale apomorphies to Gymnophthalmoidea, however, we could not identify any apomorphies that would exclude all lacertids. In these cases, we excluded lacertids because none live in North America natively, and so we instead focused on identifying tentative apomorphies or morphological differences that allowed us to differentiate between teiids, gymnophthalmids, and alopoglossids. Our cladogram helps to convey this methodology and we have updated that caption to state that it represents a “**Cladogram, pruned to only include clades that contain North American lizards, showing framework used for evaluating tentative apomorphies reported in this study based on a continentally restricted dataset.**”

The cladogram does not suggest that we did not consider snakes in our fossil identifications. We have added text stating that “We employed an apomorphy-based fossil identification framework using previously published global-scale apomorphies for squamates. Although we do not treat snake fossils here, we note that…” “...global-scale apomorphies have been evaluated in a framework that includes snakes and all fossil lizard elements described here contain apomorphic states that exclude snakes.”

Lines 132-135: We have added the following text further illuminating our methodology: “We employed an apomorphy-based fossil identification framework using previously published global-scale apomorphies for squamates… In some cases, our global-scale apomorphy based identifications allowed us to identify fossils to clades with NA and non-NA components. In these cases, we geographically restricted our identifications on the continental scale.” Any instance of failure to identify extirpated clades would be an instance of an extirpation event on a continental scale. In the late Pleistocene to Holocene these types of events are exceedingly unlikely.

With regards to how a geographically restricted dataset would affect our apomorphic framework, we note that “We supplement our global-scale apomorphic identification framework with new tentative apomorphies and morphological differences among taxa we report here based on our comparative dataset largely restricted to NA lizard taxa...” and “We note that newly reported apomorphies must be examined in non-NA taxa before being used in a global-scale apomorphic diagnosis.” Further, we note that “We were not able to examine every NA lizard species or clade and therefore take a conservative approach to fossil identifications.”

Line 141: We have added labels for relevant anatomical features on at least one specimen in each figure panel.

Line 196: We have rearranged the lettering in the panel to reflect the order mentioned in the text.

Lines 210-216: We have added labels for relevant anatomical features on at least one specimen in each figure panel.

Lines 255-257: We have added labels for relevant anatomical features on at least one specimen in each figure panel.

Lines 276-279: See our above response about not increasing the number of illustrated orientations of elements (Reviewer #1-Comment #3-Response #2).

Lines 298-299: We added a note that the posterior orbital process of the maxilla is also known as the suborbital process, which are used interchangeably in Evans (2008). Also, we have added labels for relevant anatomical features on at least one specimen in each figure panel.

Lines 310-311: We have added labels for this feature.

Line 317: We changed “wide posterior orbital process” to “medially widened posterior orbital process.”

Line 321: Even though this comment is placed on a figure panel of modern specimens we think this is in reference to the figures with fossils. We have added text in the figure captions on figures with fossils noting fossil assignment.

Lines 347-349: We have added a reference supporting our observations among teiids. We refrained from referencing too many figures that were more relevant later in the manuscript so that all the figures would not all appear together at the beginning of the manuscript.

Line 411: We have changed the wording of concluding sentences to make them more specific and reemphasize the features that permit identification.

Lines 439-448: We feel that having the specimen names in the captions as well as the figures is helpful for quickly matching and validating the specimens in the figures. It would also help readers if the species names are in the figure captions if they wished to do a quick text search for particular species. Such a search would be more difficult if the species names were only in the captions. Also, we don’t feel that there is really a need to shorten the figure captions as it would not save that much space at the cost of reducing the information contained in them.

Lines 523-524: We changed the sentence wording to “Based on these differences with other NA pleurodontans listed above, fossils were assigned to Crotaphytidae.”

Lines 639-640: We changed the sentence wording to “Based on these differences with other NA pleurodontans listed above, fossils were assigned to Crotaphytidae.”

Lines 1855-1858: Absolute horn size is variable, and what we were actually referring to was horn size relative to the size of the parietal table. In some species the horns project relatively tall compared to the size of the parietal table, while in other species, the horns barely project at all compared to the size of the table. We have changed the wording on this section to make it clear that we are actually referring to the relative size of the horns.

Line 1933: We have changed the wording on this section to make it clear that we are actually referring to the relative size of the horns.

Line 1977: We have specified the specimen letter within the figure.

Lines 2298-2303: The fossils were assigned to Anguinae and then to *Ophisaurus* with the statement on Line 2068 that “Fossils assigned to Anguinae are identified as *Ophisaurus* on the basis that *Ophisaurus* is the only genus within Anguinae known to inhabit North America during the Quaternary.” Therefore, your assessment is correct that the fossils elements are most comparable to *Ophisaurus*. However, adding panels of additional orientation is unnecessary because we clearly lay out the basis for our identification and our descriptions lay out a detailed anatomical description.

Lines 2646-2648: Yes, in Figure 36 only one specimen of *Gerrhonotus* has a notch on the posterior edge of the facial process. This is not an artifact of cropping, this is a feature that varies intraspecifically within *Gerrhonotus infernalis*. We have added additional text clearly explaining this variation and we have labeled the notch on the figures.

Lines 3262-3263. See our response to Comment 1 of Reviewer #1 regarding our justification for our decision to exclude snakes.

Line 3291: We choose to not use the term “non-snake lizards” because the term “lizard” explicitly describes a paraphyletic group and therefore does not include snakes while the clade Squamata includes lizards and snakes (de Queiroz and Gauthier 2020).

Lines 3298-3302: See our response to Comment 1 of Reviewer #1 regarding our justification for our decision to exclude snakes.

Lines 3354-3355: See our response to Comment 1 of Reviewer #1 regarding our justification for our decision to exclude snakes.

Reviewer #2

Comment 1) 1. Organization of figures—the order and reference to the figures in the manuscript is very confusing. The reader is forced to move back-and-forth amongst several figures rather than moving through them in a more logical, linear fashion. I found this to be very frustrating and often needed to set the manuscript aside out of this frustration. I believe most readers will choose to not spend the time and effort to decode this lack of organization and will therefore not get the benefits of the method and analysis that could otherwise be available. I have noted this—to the point of ad nauseum (for which I do not apologize, but I do understand that it may as equally as frustrating to the authors)—throughout the manuscript.
           Response 1) We did take careful consideration into the organization of figures. Figures are split into modern and fossil specimens and appear in the order they are referenced in the text. Within the panels of fossils, the fossils that appear together within a panel are all referred to within neighboring sections of the text. The lettering within a panel did not necessarily reflect the order in which the fossils are referred to in the text, but this would not require the reader to jump between figures and would only require one to locate the specimen within a panel. We think this is the main concern of the reviewer and we have rearranged the fossil figures so that in most cases the lettering of fossils within the panels matches the order they are mentioned in the text. In cases where the lettering of fossils within the panels does not match the order mentioned in the text this was intentionally done to keep similar fossil elements together of closely related taxa. For example, fossil squamosal identified to the *Phrynosoma douglasii* species complex is kept in the figure panel with the other fossil squamosal, which could only be identified to *Phrynosoma*. Rather than split up these fossils into two different figures, we decided that retaining fossils of the same elements both identified to *Phrynosoma* would be most helpful to readers and would not ask much of the reader.
           Response 2) In all other cases, jumping between figures is unavoidable because of the large diversity of skeletal elements from a wide variety of taxonomic groups that we illustrate. We arranged figures of modern specimens in a standardized way such that more closely related taxa are found closer together in the panel. In this way, a reader can easily look for a certain group. For example, in panels with pleurodontans, phrynosomatids always appear towards the top of the panel and corytophanids and hoplocercids always appear towards the bottom.
Comment 2) 2. Illustrated support of the methodology—The authors are presenting what they claim is an improved methodology of identifying fossil lizard taxa (why no snakes, which are just snake-lizards?) and that this method is based on the rigorous use of apomorphy-based comparisons with known extant taxa (interesting…and a bit disappointing that no attempt was made to compare to other fossil specimens from other fossil faunas). As such I was expecting an equally robust demonstration of the anatomy through figures and labelling of features. Instead, I was frustrated by the regular lack of alternative views of elements to support described features (noted often in my notes in the text). Additionally, as good as Figures 1–3 are as reference figures with comprehensive labeling of features, there are some disappointing omissions of regularly cited features (nuchal fossae, pterygoid lappet) and alternate views of elements with labeled features such as any ventral features of the frontal or parietal, lateral views of the dentary and maxilla. Additionally, the figures of fossils and representative comparative taxa (more on these below) should be labeled whenever a feature not in the Figs 1–3 reference figures are used (e.g., the “splenial spine of the dentary”). The figures of specimens represent the core data that we publish as paleontologists and if we argue for our hypotheses based on our data, then we must properly and completely illustrate that data. In the age of electronic publications, we should be able to accommodate robust and complete figures.
           Response 1) To the first point about not including snakes, our response to this critique is laid out in our response to the first comment of review #1.
           Response 2) To the second point about not comparing our sample with fossil specimens from other fossil faunas, the goal of this study was to identify the lizards from Hall’s Cave using an apomorphy based framework. Since our identifications are rarely below the genus level, a comparison with other fossil faunas would likely have no effect in this goal particularly for the late Quaternary.
           Response 3) To the third point regarding lack of alternative views of elements, this is laid out in our second response to comment 3 of reviewer #1, but we will further elaborate here since additional points have been brought up on the topic. We agree that illustrating anatomy is important in paleontological studies and all views of a fossil are relevant, but in most cases we are limited in our ability to present a 3D object onto a 2D medium. In many of our fossil descriptions and identifications at least three different views of fossils contain relevant morphology for identification; however, illustrating all these views would double or triple the number of figures which is already close to 50. Doing the same for modern specimens would add an even greater number of figures. We therefore struck a balance and only included views of the most important standard anatomical views of elements. We feel it is largely unnecessary to add additional orientations for the following reasons. Reason 1): In the line edits, the reviewer mentions that figures are data which we agree with; however, we disagree that they are necessary to “confirm or reject the interpretation.” Figures are one way to illustrate the relevant morphology; however, descriptions are also fundamental data and are sufficient for replicability to confirm or reject interpretations. Reason 2) We examined a comparative dataset of over 90 species of North American lizards, all of which provided core morphological data that was important for our fossil identifications, yet we are limited in what we can reasonably present in this manuscript. The replicability in our study lies in the fact that we explicitly lay out detailed descriptions of the fossils paired with the criteria by which we are identifying fossils. Further, these fossils and comparative specimens are curated in a public institution and are available for reexamination.
           Response 4) To the point that figures should be labeled when a morphological feature is not labeled in the reference figures, we agree, and we have added additional labels on at least a few specimens in each panel to highlight relevant morphology.
Comment 3) 3. Reference/comparative anatomy images—At the risk of having egg on my face regarding the previous sentence I want to discuss what at times felt like the overuse of comparative figures. While useful and supportive of the apomorphic comparative identifications (what we used to just call comparative anatomy although here without—understandably—the differential aspect of a differential diagnosis), the comparative images greatly lengthen the manuscript. If the addition of these figures is considered essential to the methodology (and I would agree that they are) I wonder if there is a better balance? Fewer elements (though I appreciate the comprehensive nature of the images and I do believe they add to the overall value of the manuscript as an osteological resource)? Move them to the SI (though that brings back the concern of the flow of images relative to the references in the text)? In short, I bring this up based on my editorial concerns, but I do feel that these images are important and add value to the manuscript beyond the methodology and updating of the fauna.
           Response 1) Above, we agreed with the reviewer that the figures are core data to the manuscript, therefore, we feel it would be inappropriate to move the figures to supplemental data. The number of figured elements is exactly the number of the different types of lizard cranial elements encountered from Hall’s Cave therefore, we do not feel it necessary to reduce the number of elements presented.
Comment 4) 4. Anatomy errors—Thankfully these are few and the authors have done a very good job in managing the descriptions of complex anatomy. However, the superior alveolar foramen of the maxilla CANNOT be medial to the palatine process as that is the most medial feature of the maxilla. The foramen is lateral to the palatine process and the palatine is medial to the palatine process. The maxilla (derivative 1a of arch 1) conducts the maxillary neurvascular bundle consisting of the V2 nerve, maxillary artery, and maxillary vein(s). This system of passages and contents is knows as the superior alveolar system and all reference to openings (posterior superior alveolar foramen lateral to the palatine process, anterior superior alveolar foramen typically just posterior the premaxillary process, nutrient foramina along the lateral surface) all must be identified specifically and accurately. The same is true for the distribution in the dentary/mandible. The V3 nerve and mandibular/inferior alveolar AV are the equivalent distribution systems of derivative 1b of arch 1. As such all identification must be specific and accurate as part of the inferior alveolar system.
           Response 1) The description of the position of the superior alveolar foramen of the maxilla has been changed and now places it lateral to the palatine process.
           Response 2) The terminology for the superior alveolar system has been corrected. We now refer to the foramina on the premaxillary process of the maxilla as openinging for the superior alveolar canal and subnarial arterial foramen following Smith (2009) “Eocene Lizards of the Clade Geiseltaliellus from Messel and Geiseltal, Germany, and the Early Radiation of Iguanidae (Reptilia: Squamata)” Bulletin of the Peabody Museum of Natural History, 50(2), 219–306.
Comment 5) 5. Anatomical absences—I am curious as to why no vertebrae or limb elements were described or figured. Is that to be another project? Additionally, as mentioned above, there must have been substantial numbers of snake vertebrae and at least some snake cranial elements. Where are they in a paper on squamates? 6. Citations at end of manuscript—In the interest of time, I did not fully check all the references, but I did note that the authors did not use a single formatting strategy for all citations at the end of the manuscript and I encourage them to follow the PlosOne formatting directions for citations.
           Response 1) Fossil squamate postcranial elements are present in Hall’s Cave; however, postcranial elements are much more difficult to identify to low taxonomic levels. The goal of this study was to examine the diversity of lizards from Hall’s Cave and in this case identification of postcranial material would not have added any information since postcranial material could only be identified at higher taxonomic levels compared to cranial elements. Furthermore, patterns of variation in the postcranial skeleton of lizards are poorly studied and an adequate examination of variation in fossils and modern taxa would require an equal or greater treatment of detailed anatomy as presented in this current study on the skull.
           Response 2) To the point about not including snakes, our response to this critique is laid out in our response to the first comment of review #1.
           Response 3) We reviewed and standardized our citation formats to a single style.

Line 45: We replaced the hyphen with an en dash.

Line 125: In many cases, our global-scale apomorphy based identifications allowed us to identify fossils to clades with NA and non-NA components. In these cases, we geographically restricted our fossil identifications using tentative apomorphies restricted on the continental scale. Our cladogram helps to convey this methodology and we have updated that caption to state that it represents a “**Cladogram, pruned to only include clades that contain North American lizards, showing framework used for evaluating tentative apomorphies reported in this study based on a continentally restricted dataset.**”

Line 196: We did take careful consideration into the organization of figures. Figures are split into modern and fossil specimens as well as by taxonomy. Pleurodontan fossils are described and illustrated first. Within the figures of fossils, the fossils that appear together within a panel are all referred to within neighboring sections of the text. The lettering within a panel did not necessarily reflect the order in which the fossils are referred to in the text, but this would not require the reader to jump between figures and would only require one to locate the specimen within a panel. We think this is the main concern of the reviewer and we have rearranged the fossil figures so that lettering of fossils within the panels matched the order mentioned in the text.

In all other cases, jumping between figures is unavoidable because of the large diversity of skeletal elements from a wide variety of taxonomic groups that we illustrate. We arranged figures of modern specimens in a standardized way such that more closely related taxa are found closer together in the panel. In this way, a reader can easily look for a certain group. For example, in panels with pleurodontans, phrynosomatids always appear towards the top of the panel and corytophanids and hoplocercids always appear towards the bottom.

Line 202: See our Response 3 to Comment 2 of Review #2 regarding alternative views of elements.

Line 306: We have changed our wording to better reflect the morphological difference we are describing. The sentence now reads “A well-defined depression is absent in non-iguanian lizards…”

Lines 340-343: See our Response 3 to Comment 2 of Review #2 regarding alternative views of elements.

Line 397: See our Response 3 to Comment 2 of Review #2 regarding alternative views of elements.

Line 433: See our Response 3 to Comment 2 of Review #2 regarding alternative views of elements.

Line 476: Changed “lacks” to “lack.”

Line 499: We have rearranged the order of fossils in panels so that lettering of fossils within the panels matched the order mentioned in the text.

Line 561: Our understanding of the morphology of *Laemanctus longipes* comes from descriptions and figures in the literature. We have added additional text here making this clear. Furthermore, in our methods we have a statement that in cases where we lacked comparative material on hand “we … relied on morphological evidence from the published literature.”

Line 658: We have rearranged the order of fossils in panels so that lettering of fossils within the panels matched the order mentioned in the text.

Line 704: Changed “Pterygoids” to “fossil pterygoids.”

Line 742: Changed “slendered” to “more slender.”

Line 790: See our Response 3 to Comment 2 of Review #2 regarding alternative views of elements.

Lines 973-975: The terminology for the superior alveolar system has been corrected.

Lines 1047-1048: See our Response 3 to Comment 2 of Review #2 regarding alternative views of elements.

Lines 1054: See our Response 3 to Comment 2 of Review #2 regarding alternative views of elements.

Line 1071: See our Response 3 to Comment 2 of Review #2 regarding alternative views of elements.

Line 1217: We have now included a comment about the difference in the posterior extent of the intramandibular septum. For the second part of the comment, see our Response 3 to Comment 2 of Review #2 regarding alternative views of elements.

Line 1265: We have rearranged the order of fossils in panels so that lettering of fossils within the panels matched the order mentioned in the text.

Line 1267: See our Response 3 to Comment 2 of Review #2 regarding alternative views of elements.

Line 1461: We have rearranged the order of fossils in panels so that lettering of fossils within the panels matched the order mentioned in the text.

Line 1466: See our Response 3 to Comment 2 of Review #2 regarding alternative views of elements.

Line 1467: We have rearranged the order of fossils in panels so that lettering of fossils within the panels matched the order mentioned in the text.

Line 1492: We have rearranged the order of fossils in panels so that lettering of fossils within the panels matched the order mentioned in the text.

Line 1518: Changed “Fossils” to “Fossil.”

Line 1584: The terminology for the superior alveolar system has been corrected.

Lines 1586-1587: See our Response 3 to Comment 2 of Review #2 regarding alternative views of elements.

Line 1806: See our Response 3 to Comment 2 of Review #2 regarding alternative views of elements.

Line 1831: We have rearranged the order of fossils in panels so that lettering of fossils within the panels matched the order mentioned in the text.

Line 1850: We have rearranged the order of fossils in panels so that lettering of fossils within the panels matched the order mentioned in the text.

Line 1867: We have rearranged the order of fossils in panels so that lettering of fossils within the panels matched the order mentioned in the text.

Line 1893: We have rearranged the order of fossils in panels so that lettering of fossils within the panels matched the order mentioned in the text.

Lines 1924-1925: See our Response 3 to Comment 2 of Review #2 regarding alternative views of elements.

Line 1959: In this case, the fossil squamosal identified to the *Phrynosoma douglasii* species complex is kept in figure 29 because the other fossil squamosal, which could only be identified to *Phrynosoma*, appears in figure 29. Rather than split up these fossils into two different figures, we decided that retaining fossils of the same elements both identified to *Phrynosoma* would be most helpful to readers. This would only require the reader to refer to one figure prior.

Line 1977: “E” added to figure citation.

Line 2042: “C” added to figure citation.

Lines 2084-2085: See our Response 3 to Comment 2 of Review #2 regarding alternative views of elements.

Line 2124: See our Response 3 to Comment 2 of Review #2 regarding alternative views of elements. This applies to described microscopic features as well.

Line 2132-2133: The terminology for the superior alveolar system has been corrected.

Lines 2178-2179: See our Response 3 to Comment 2 of Review #2 regarding alternative views of elements.

Line 2218: See our Response 3 to Comment 2 of Review #2 regarding alternative views of elements.

Line 2222: See our Response 3 to Comment 2 of Review #2 regarding alternative views of elements.

Lines 2337-2338: See our Response 3 to Comment 2 of Review #2 regarding alternative views of elements.

Line 2380: In this instance, the location of the feature we are describing is most easily referred to based on the position of muscle attachment. By indicating that the feature we are describing is associated with location of a muscle attached, readers can better discern the particular morphology we are describing.

Line 2417: The point about the subdental parapet is an interesting one. We looked into this and based on our search, we found that there are indeed some patterns in the presence of a subdental gutter; however, there seems to be no universal agreement on what constitutes a subdental parapet and patterns on the presence of a subdental gutter don’t seem to be as useful for identifying and distinguishing between anguimorph taxa as was suggested by the reviewer. A subdental gutter, defined as a “conspicuous groove lingual to the tooth row” (Evans 2008, pg. 273), is reportedly present in geckos, xantusiids, and cordylids. A subdental shelf, defined as a “medial projection from the alveolar margin” (Evans 2008, pg. 273), is reportedly small in iguanians, anguimorphs, amphisbaenians, and some gymnophthalmids (Estes et al. 1988). A subdental ridge, defined as a thickened ventral region of the subdental shelf (Evans 2008), is small in most iguanians and anguimorphs (except for *Diploglossus* and *Xenosaurus*), but is developed in “scincids, cordyliforms, some teiids, and acrodont lizards, but is shallower in lacertids, most teiids, and gymnophthalmids” (Evans 2008, pg. 273). We are inclined to think that the terms subdental ridge, subdental parapet, and suprameckelian lamina have been used interchangeably in the literature although there are important distinctions. A subdental parapet seemingly describes a dorsal expansion of the subdental ridge which result in the formation of a subdental gutter (as used by Gauthier 1982); however, the presence of a subdental ridge or suprameckelian lamina in and of itself is not enough to form a subdental gutter. The formation of a subdental gutter; however, need not be caused by a dorsal expansion of the subdental ridge since it could also be a result of a ventral shifting of the alveolar margin.

The subdental shelf and gutter morphologies were considered an ordered character by Gauthier et al. (2012) and individuals that had no subdental shelf or a weakly developed subdental shelf necessarily lacked a subdental gutter (Gauthier et al. 2012, character 360). Even though a subdental gutter is reportedly absent among anguimorphs, it was reported as present in *Aniella* by Gauthier (1982), albeit the gutter is more broad and shallow compared to the gutter found in some other squamates.

With regards to the terminology for the splenial spine, we did not come up with the term splenial spine and instead are following an existing terminology already used in many papers describing fossil and modern lizard anatomy (e.g. Roček 1980 “The dentition of the European glass lizard *Ophisaurus apodus* (Pallas, 1775)(Reptilia, Sauria: Anguidae), with notes on the pattern of tooth replacement”; Čerňanský et al. 2014 “Additions to the lizard fauna (Squamata: Lacertilia) of the upper Oligocene (MP 28) of Herrlingen 8, Southern Germany”; Klembara et al. 2014 “Comparative Anatomy of the Lower Jaw and Dentition of *Pseudopus apodus* and the Interrelationships of Species of Subfamily Anguinae (Anguimorpha, Anguidae)”). Since the reviewer did not offer an alternative term and since the term provides a clear way for us to refer to this anatomical feature we are inclined to retain the terminology we have used.

Line 2524: In this case, we chose to leave TxVP 41229-27659 in figure 31 because we thought it more important for all fossils referred to *Ophisaurus* appear in a figure together and the only three elements could be identified to *Gerrhonotus* did not need their own figure. In this case, we chose to group the few fossils identified to *Gerrhonotus* or Anguidae together to reduce the number of figures. We felt that asking readers to refer back to a previous figure was fine in this case.

Line 2529: See our above response (regarding the comment in Line 2417) about the subdental parapet and subdental gutter.

Lines 2535-2535: We have further clarified the relationship between the splenial spine and the anterior inferior alveolar foramen. The relationship between the spaniel spine and the anterior inferior alveolar foramen was also noted previously in lines 2435-2437.

Line 2544: See our above response regarding the comment in Line 2524.

Lines 2625-2628: See our Response 3 to Comment 2 of Review #2 regarding alternative views of elements.

Lines 2632-2633: The terminology for the superior alveolar system has been corrected.

Lines 2701-2702: See our Response 3 to Comment 2 of Review #2 regarding alternative views of elements.

Line 2736: The terminology for the superior alveolar system has been corrected.

Lines 2763-2765: See our Response 3 to Comment 2 of Review #2 regarding alternative views of elements.

Line 2783: We agree the nuchal fossae are well-developed and we have changed our wording to reflect this.

Lines 2786-2789: See our Response 3 to Comment 2 of Review #2 regarding alternative views of elements.

Line 2847: Figure citation added.

Line 2869: We agree that the pterygoid lappet is very small, but we don’t think it is absent. We have changed our description to “minute pterygoid lappet” to better reflect this.

Lines 2897-2898: See our Response 3 to Comment 2 of Review #2 regarding alternative views of elements.

Lines 2925-2927: See our Response 3 to Comment 2 of Review #2 regarding alternative views of elements.

Lines 2937-2938: See our Response 3 to Comment 2 of Review #2 regarding alternative views of elements.

Lines 2977-2978: See our Response 3 to Comment 2 of Review #2 regarding alternative views of elements.

Lines 3042-3043: See our Response 3 to Comment 2 of Review #2 regarding alternative views of elements.

Line 3065: See our Response 3 to Comment 2 of Review #2 regarding alternative views of elements.

Line 3069: “wavy” changed to “undulating”

Line 3071: See our Response 3 to Comment 2 of Review #2 regarding alternative views of elements.

Line 3091: We have removed the parenthetical and reference the nuchal fossae directly.

Line 3093: See our Response 3 to Comment 2 of Review #2 regarding alternative views of elements.

Line 3160-3161: See our Response 3 to Comment 2 of Review #2 regarding alternative views of elements.

Lines 3163-3164: See our Response 3 to Comment 2 of Review #2 regarding alternative views of elements.

Line 3185: We chose not to use a citation to discuss the potential tooth erosion patterns. This is actually a topic for a follow-up study using an experimental framework developed by Smith et al. (2021) “A model of digestive tooth corrosion in lizards: experimental tests and taphonomic implications” Scientific Reports, 11:12877.
