# Supplementary material for: Identification of Late Pleistocene and Holocene fossil lizards from Hall’s Cave (Kerr County, Texas) and a primer on morphological variation in North American lizard skulls
Source: PLoS One. 2024 Aug 15;19(8):e0308714. doi: 10.1371/journal.pone.0308714 (PMC11326655; doi:10.1371/journal.pone.0308714)
Supplement: Attachment — Submitted filename: Response to Reviewer.docx [file pone.0308714.s009.docx]

Reviewer #1

Comment 1. The authors are using cladistic methods while trying to maintain traditional groupings (i.e., squamate apomorphies with a paraphyletic "lizard" concept, distinct from snakes). I see issues with this method as apomorphies are determined from cladistic groupings, and due to snakes being deeply nested within squamates, the classical definition of "lizards" and snakes becomes problematic. I would like to see further explanation of how this is being addressed. The phylogeny being presented excludes snakes, but is stated to be derived from a study that includes all squamates. The apomorphies thus constructed from the provided phylogeny are somewhat artificial without the consideration of potential character overlap with snakes. This can be further exemplified when assessing apomorphies of closely related clades, such as anguids that may share apomorphic states with snakes. Expansion in the methods of how the authors are accounting for this, or mention of the potential shortcomings of their methods in light of snakes deeply nested position within squamates should be included.

Response 1. Based on this comment we think there may be a misunderstanding of our methodology. To be clear- our apomorphic identifications explicitly account for snakes, even though we did not describe any fossil snakes. None of the apomorphies that we used from the literature or developed here diagnose “lizards,” they diagnose specific clades within Squamata that are universally called lizards (e.g., pleurodontan iguanians, teiids, gerrhonotines, etc.). Our decision to treat only fossil lizards and not snakes is a practical one and does not reflect the systematic framework or apomorphic identification methodology. As stated previously we decided to limit this paper to lizards to allow a tractable manuscript length, and because snakes are usually treated apart from other squamates in herpetological studies of both extant and extinct taxa (even though “lizards” is a paraphyletic group). This extends to the cladogram that was previously presented; it was pruned to only include the taxa being studied, and did not indicate that only taxa in the cladogram were included in the apomorphic diagnoses. In sum, in this study we take apomorphies from the literature that have been evaluated for all squamates (including snakes and lizards)-when we identify fossils to particular clades using these apomorphies we are explicitly taking snakes into account.

We have revised our wording in our methods section to clarify our methodology (lines 137 - 144). We also updated the cladogram that we present in the manuscript to include squamates globally while highlighting the North American lizard clades to better convey our identification framework.

Comment 2. Additionally, when features are mentioned in the text that are key to identifying fossils to a living clade, those features should be labelled in one or more of the figures. I noticed several features such as the palatal shelf of the premaxilla, and the pterygoid facet of the quadrate were not labelled in any figure, to name a few. Review of key features and addition of appropriate labels should be done as well.

Response 1. We have added labels in the figures for the palatal shelf of the premaxilla and the pterygoid facet of the quadrate. We have also reviewed the manuscript to ensure that those key morphological features are labeled. We also note that because we state in our methods that our anatomical terminology follows that of Evans (2008) “The skull of lizards and Tuatara”, readers unfamiliar with terminology used in our manuscript can reference that comprehensive work as well as the literature we reference throughout the manuscript for clarification.

Reviewer #2

We thank the reviewer for their kind words regarding our study. We do hope that our research will assist paleontologists working on Quaternary fossil sites with squamate remains and spark new interest among researchers into patterns of osteological variation in extant squamates.
